# Erratum

**Published:** 2005-09

In "Decrease in Anogenital Distance among Male Infants with Prenatal Phthalate Exposure" [ Environ Health Perspect 113:1056–1061 (2005)], Shanna Swan’s affiliation was listed as University of Rochester, Rochester, Minnesota. The correct location is Rochester, New York.

*EHP* regrets the error.

